# Metabolic syndrome is associated with better prognosis in patients with tongue squamous cell carcinoma

**DOI:** 10.1186/s40880-015-0009-7

**Published:** 2015-04-08

**Authors:** Lan Zou, Tian-Run Liu, An-Kui Yang

**Affiliations:** Department of Head and Neck Surgery, Sun Yat-sen University Cancer Center; State Key Laboratory of Oncology in South China; Collaborative Innovation Center for Cancer Medicine, Guangzhou, Guangdong 510060 P. R. China; Department of Otorhinolaryngology Head and Neck Surgery, The Sixth Affiliated Hospital of Sun Yat-sen University, Guangzhou, Guangdong 510655 P. R. China

**Keywords:** Metabolic syndrome, Tongue squamous cell carcinoma, Prognosis

## Abstract

**Introduction:**

Metabolic syndrome (MS) is associated with several cancers, but it is not clear whether MS affects the prognosis of tongue squamous cell carcinoma (TSCC). This study aimed to evaluate the prognostic value of MS in TSCC.

**Methods:**

Clinical data from 252 patients with TSCC who were initially treated at the Sun Yat-sen University Cancer Center between April 1998 and June 2011 were collected, and the associations between MS and clinicopathologic factors were retrospectively analyzed. Prognostic outcomes were examined by Kaplan-Meier analysis and Cox regression analysis.

**Results:**

Of the 252 patients, 48 were diagnosed with MS. MS was associated with early N category in TSCC (*P* < 0.001). The patients with MS showed longer survival than those without MS (*P* = 0.028). MS was an independent prognostic factor for patients with TSCC.

**Conclusions:**

MS is associated with early N category in TSCC. It is an independent prognostic factor for better survival in patients with TSCC.

## Background

Oral squamous cell carcinoma (OSCC) is a common malignant tumor worldwide. OSCC accounts for over 90% of all oral cancers [1], and the most common location for this disease is the tongue [2]. Tongue squamous cell carcinoma (TSCC) is especially prevalent in low-income communities in North France, East Europe, South America, and Southeast Asia, and 90% of patients with TSCC are over 45 years old [3]. TSCC seriously affects quality of life of the patients and carries a poor prognosis, with a 5-year overall survival (OS) rate of 56% [4].

Metabolic syndrome (MS) is a cluster of metabolic abnormalities. The pathophysiologic basis of MS is insulin resistance [5]. The diagnosis criteria for MS include central obesity, hyperglycemia, hypertriglyceridemia, hypertension, and low serum concentration of high-density lipoprotein (HDL) [6]. MS is related to several cancers, including breast cancer, prostate cancer, and gastric cancer. MS and its components are associated with worse survival in breast cancer [7] and prostate cancer [8], but better survival for gastric cancer [9]. Obesity was reported to be an adverse independent prognostic factor for early-stage TSCC [10]. However, there is little information regarding the association between MS and TSCC or the impact of MS on TSCC patient survival. In this study, we tried to analyze whether the status of MS before treatment have any impact on the OS in patients with TSCC.

## Methods

### Patient selection

A total of 252 patients diagnosed with TSCC who were initially treated at the Sun Yat-sen University Cancer Center between April 1998 and June 2011 were involved in this study, including 145 males (58%) and 107 females (42%). Subject ages ranged from 20 to 89 years, with a median age of 52 years. None of these patients had distant metastasis before treatment. All patients underwent surgery; 59 (23%) underwent multimodality therapy including surgery. Surgical margins were tumor-free for all patients. The data regarding MS and its components were recorded before treatment. The OS was defined as the duration from the date of initial treatment to the date of death or the last follow-up (July 2014). This study followed the Declaration of Helsinki for medical protocol and ethics. Study approval was obtained from independent ethics committees at Cancer Center of Sun Yat-Sen University.

### Diagnosis criteria of MS

According to the National Cholesterol Education Program’s Adult Treatment Panel III, our diagnosis criteria of MS included (1) fasting plasma glucose (GLU) ≥6.1 mmol/L or a diagnosis of diabetes; (2) abdominal obesity, with a body mass index (BMI) ≥25 kg/m^2^; (3) triglycerides (TG) ≥1.7 mmol/L; (4) high-density lipoprotein (HDL) ≤1.04 mmol/L for males and ≤1.3 mmol/L for females; and (5) hypertension, with the systolic blood pressure (BP) ≥130/80 mmHg. Meeting 3 or more of the criteria was necessary for diagnosis [9].

### Statistical analyses

The chi-square test was performed to analyze the relationship between MS and clinicopathologic factors of TSCC. Kaplan-Meier and log-rank tests were used for survival analysis. Multivariate Cox regression analysis was performed for significant variables identified by using univariate analysis. SPSS 16.0 software was used for all analyses. A *P* value of <0.05 was considered statistically significant.

## Results

### Association between MS and clinicopathologic characteristics of TSCC

The clinicopathologic characteristics of 252 patients are shown in Table 1. Of these patients, 48 (19.1%) were diagnosed with MS. MS was associated with early N category (*P* < 0.001). However, there was no association between MS and age, gender, T stage, pathologic grade, treatment strategy, or tumor location.

**Table 1:** **Association between MS and clinicopathologic characteristics of patients with TSCC**

**Characteristic**	**Number of patients (%)**			***P*** **value**
	**Total**	**With MS**	**Without MS**	
Age (years)				0.426
≥52	134 (53)	28 (58)	106 (52)	
<52	118 (47)	20 (42)	98 (48)	
Sex				0.273
Male	145 (58)	31 (65)	114 (56)	
Female	107 (42)	17 (35)	90 (44)	
T category				0.524
T1/T2	222 (88)	41 (85)	181 (89)	
T3/T4	30 (12)	7 (15)	23 (11)	
N category				<0.001
N0	130 (52)	36 (75)	94 (46)	
N+	122 (48)	12 (25)	110 (54)	
Pathologic grade				0.598
Well differentiated	184 (73)	33 (69)	151 (74)	
Moderately differentiated	58 (23)	12 (25)	46 (23)	
Poorly differentiated	10 (4)	3 (6)	7 (3)	
Treatment				0.639
Surgery alone	193 (77)	38 (79)	155 (76)	
Multimodality therapy	59 (23)	10 (21)	49 (24)	
Tumor location				0.653
Lateral margin of the tongue	185 (73)	34 (71)	151 (74)	
Other locations^a^	67 (27)	14 (29)	53 (26)	
Total	252	48	204	

MS, metabolic syndrome; TSCC, tongue squamous cell carcinoma. ^a^Other locations here include the apex linguae, dorsum, and ventrum of the tongue.

### Relationship between MS and survival of TSCC patients

**Figure 1:**
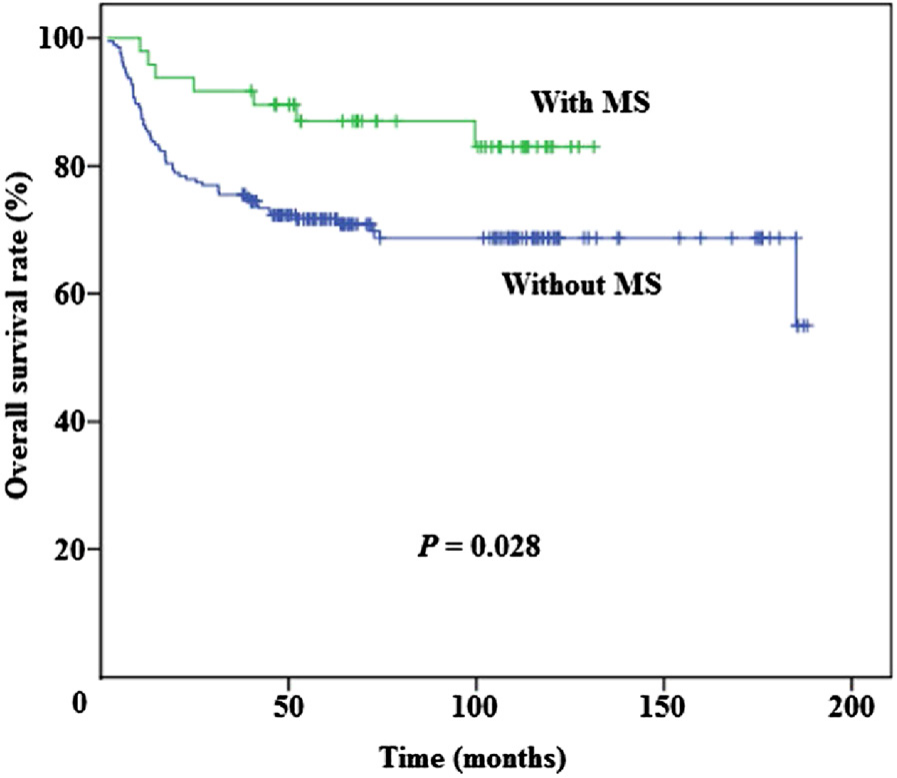
**Overall survival curves for patients with tongue squamous cell carcinoma (TSCC) according to the status of metabolic syndrome (MS).** Kaplan-Meier analysis results indicate that patients with MS have better overall survival than those without MS (*P* = 0.028).

At the time of the last follow-up, 184 patients (73.0%) were alive, and 68 (27.0%) died of cancer-related diseases. Figure 1 demonstrates that the patients with MS had better OS than those without MS (*P* = 0.028). The Cox proportional hazards model was used to verify whether MS and other variables were independent prognostic factors for TSCC patients. The univariate analysis results showed that MS, sex, age, T category, N category, pathologic grade, and treatment strategy were associated with OS. Multivariate Cox regression analysis revealed that MS, age, T category, N category, pathologic grade, and treatment strategy were independent prognostic factors for patients with TSCC (Table 2).

**Table 2:** **Univariate and multivariate Cox regression analyses for overall survival in patients with TSCC**

**Variable**	**Univariate analysis**		**Multivariate analysis**	
	**HR (95% CI)**	***P*** **value**	**HR (95% CI)**	***P*** **value**
Sex^a^	0.591 (0.356–0.984)	0.043	0.681 (0.398–1.166)	0.162
Age (years)				
<52	Ref		Ref	
≥52	1.823 (1.100–3.020)	0.020	2.149 (1.279–3.614)	0.004
Tumor location^b^	1.032 (0.607–1.754)	0.908		
Pathologic grade				
Well differentiated	Ref		Ref	
Moderately differentiated	2.483 (1.475–4.183)	0.001	2.417 (1.417–4.124)	0.001
Poorly differentiated	4.728 (2.101–10.639)	<0.001	2.726 (1.172–6.336)	0.020
T category				
T1/T2	Ref		Ref	
T3/T4	3.575 (2.054–6.223)	<0.001	2.007 (1.091–3.691)	0.025
N category				
N0	Ref		Ref	
N+	4.846 (2.725–8.618)	<0.001	3.408 (1.873–6.202)	<0.001
Treatment ^c^	3.963 (2.451–6.407)	<0.001	2.559 (1.492–4.392)	0.001
MS				
With MS	Ref		Ref	
Without MS	2.342 (1.070–5.125)	0.033	2.518 (1.126–5.631)	0.024
One component meets the criteria of MS				
BMI	0.692 (0.330–1.448)	0.328		
GLU	0.938 (0.491–1.792)	0.846		
BP	0.745 (0.455–1.221)	0.243		
TG	0.695 (0.391–1.235)	0.215		
HDL	0.818 (0.491–1.361)	0.439		

^a^Male versus female; ^b^the margo lateralis linguae versus other locations (the apex linguae, dorsum, and ventrum of the tongue); ^c^surgery alone versus multimodality therapy. TSCC, tongue squamous cell carcinoma; HR, hazard ratio; CI, confidence interval; Ref, reference; MS, metabolic syndrome; BMI, body mass index; GLU, fasting plasma glucose; BP, blood pressure; TG, triglycerides; HDL, high-density lipoprotein.

## Discussion

Our study demonstrated that MS was associated with early N category in TSCC. In addition, MS was an independent prognostic factor for better survival in patients with TSCC.

This study first reported the association of MS with early N category in TSCC. Previous reports demonstrated that MS was associated with better differentiation in gastric cancer cells [9], whereas others indicated that MS or its components were associated with a more aggressive tumor type in colon cancer and prostate cancer [11,12]; the influence of MS on breast cancer remains controversial [13,14]. The mechanism by which MS influences N category is not well understood. Insulin receptor and insulin-like growth factor 1 (IGF-1) are expressed in most cancer cells, and IGF-1 can stimulate invasion and proliferation of cervical cancer cells [15]. The patients with diabetes had low concentrations of IGF-1 [16]. Moreover, the insulin receptor-activating signaling pathways may offer protection from invasion and metastasis of cancer cells [17,18]; however, the mechanism remains unclear and requires further investigation. In addition, underweight patients in China were associated with lower income and education, and they were less likely to receive the correct treatment when diagnosed with an early-stage disease. The TNM classification significantly affects tongue cancer prognosis; the earlier the classification, the better the prognosis. In our study, T category and N category were independent prognostic factors for patients with TSCC. Liu *et al.* [19] reported that the 5-year OS rates for patients with stages I, II, III, and IV oral cancers were 79.8%, 68.2%, 57.2%, and 50.4%, respectively. Thus, it is critical to diagnose tongue cancer in early stages [3]. In addition, the pathologic grade significantly affects prognosis of the patients. The patients who underwent multimodality therapy in our study usually had late-stage disease, which leaded to worse prognosis of these patients.

The impact of MS on cancer patient prognosis, including cervical cancer, remains controversial. For example, MS predicts poor survival in patients with prostate cancer and breast cancer [7,8], whereas Wei *et al.* [9] reported that old patients with early-stage gastric cancer and MS had a better prognosis. In patients with early-stage colon cancer, diabetes and hypertension predicted poor survival, but dyslipidemia predicted good survival [20,21]. Another study reported increased odds of MS among American women with a history of cervical cancer, but no association was observed between the single component of MS and cervical cancer [22]. Several reports suggested that leanness might be associated with poor outcome for patients with cervical cancer [19,23-26]. However, obesity was considered an independent predictor of increased risk of death in patients with early-stage tongue cancer [10]. However, patients in these studies had various tumor sites and different pathologic diagnoses. In addition, some of these studies were limited by small sample sizes. Our study found that MS was associated with better prognosis in patients with TSCC. Malnutrition is common in patients with head and neck cancer, especially in oral tongue cancer, which seriously affects the patient’s nutritional status, so weight loss before treatment was associated with poor prognosis. In addition, patients without MS are more likely to suffer from nutritional deficiency, which may lead to poor prognosis. Good nutritional status could improve survival by strengthening immunity and providing high tolerance for lengthy therapeutic periods. A retrospective research of oral cavity cancer and oropharyngeal cancer showed that weight loss was a strong predictor of death [19,24,26]. Another study also reported poor survival in oral cancer patients with a BMI < 22.8 kg/m^2^ before surgery [19].

Several individual components of MS have been recognized as carcinogenic. However, our research did not identify any significant influence of MS components on TSCC prognosis. This result was similar to those of previous studies [9,22]. Epidemiologic studies indicated that clustering MS components increased the carcinogenic effect on colorectal cancer development and mortality compared with individual factors [27,28]. Our findings align with this theory of synergism among MS components, as none of the individual MS components was associated with TSCC, but when clustering at least 3 components, this association became significant and remained significant when adjusting for other risk factors for TSCC. Further investigations are needed to better understand the effects of MS and its components on survival in TSCC patients.

The molecular and cellular mechanisms by which MS affects cancer patient survival are very complicated. Smith *et al.* [29] reported that IGF-1 is a potential pathway linking the environment with cancer. High levels of IGF-1 increases the risk of cancer and aggressiveness of malignancies. Cowey *et al.* [30] reported that TG promoted cancer cell proliferation and showed anti-apoptotic activity due to the generation of reactive oxygen species (ROS) and oxidative stress, which cause DNA damage. Obesity is linked with higher incidence and mortality of several cancers, but there are still some opposing opinions [10,19,23,24,26]. Levels of adipokine, leptin, and adiponectin in obesity can affect several signal transduction pathways involved in cell survival [31], and Shin *et al.* [32] reported that the adiponectin receptor is related to gastric cancer development, progression, and poor survival. BMI influences cancers by releasing several inflammatory mediators, such as tumor necrosis factor alpha, interleukin-6, and prostaglandin E2 [33]. A previous study reported an association between cervical cancer and low HDL levels [22].

Our study had some limitations. First, it was a retrospective study. Second, this study did not include disease-free survival due to limited clinical data. Third, we did not know the exact time of the occurrence of MS, which may lead to an overestimation of the associations.

## Conclusions

In conclusion, this study first demonstrated that MS is associated with early N category and predicts good prognosis in patients with TSCC. Understanding the underlie molecular and cellular mechanisms may provide clues to prevent cancer development. Similarly, therapeutic interventions targeting these molecular mechanisms might manifest a positive perspective for the treatment of TSCC. In addition, our results indicate that good nutritional status may improve survival in patients with TSCC.

## Consent

Written informed consent was obtained from the patient for the publication of this report and any accompanying images.
